# The effects of competitive trait anxiety on attentional bias in adolescent tennis players

**DOI:** 10.3389/fpsyg.2026.1773144

**Published:** 2026-03-30

**Authors:** Xiaozhong Su, Rong Shangguan, Meilian Chen

**Affiliations:** 1 School of Educational Sciences, Hunan Normal University, Changsha, China; 2 School of Sports and Music, Central South University of Forestry and Technology, Changsha, China; 3 College of Physical Education, Hunan Normal University, Changsha, China; 4 Public Course Department of Changde Vocational and Technical College, Changde, China

**Keywords:** adolescent tennis players, attentional bias, competitive trait anxiety, difficulty disengaging, dot-probe task, emotional faces, social expectation anxiety

## Abstract

**Background:**

Competitive anxiety is common in adolescent athletes and may bias the processing of socio-emotional cues in competition settings. However, evidence linking competitive trait anxiety to specific attentional-bias components in adolescent tennis players remains limited. This study examined group characteristics of competitive trait anxiety and tested whether athletes with different anxiety levels show distinct attentional-bias patterns toward emotional faces.

**Methods:**

A total of 120 adolescent tennis players (aged 14–18 years) who participated in the 2020 Hunan Provincial Youth Tennis Championship completed the Pre-competition Emotion Scale–Trait (PES-T). Athletes scoring in the top and bottom 20% were selected to form a high-anxiety group (*n* = 24) and a low-anxiety group (*n* = 24). Using positive, negative, and neutral faces selected from the Chinese Affective Face Picture System, participants completed a modified dot-probe task. Indices of attentional orienting and difficulty disengaging from emotional cues were computed. Correlation and regression analyses were conducted between anxiety dimensions and attentional-bias indices.

**Results:**

(1) Female athletes reported significantly higher competitive trait anxiety than males. (2) Competitive trait anxiety tended to decrease with greater age, longer training experience, and higher sport level. (3) The high-anxiety group showed a pronounced difficulty disengaging from negative faces, indicating a negative attentional bias; the low-anxiety group showed a significant bias toward positive faces.(4)Within the high-anxiety group, social expectation anxiety was positively associated with, and significantly predicted, difficulty disengaging from negative cues.

**Conclusion:**

Competitive trait anxiety in adolescent tennis players is shaped by gender and training experience and may influence cognitive resource allocation by biasing attention to emotional information—especially by prolonging engagement with negative cues. Social expectation anxiety appears to be a key risk factor for negative disengagement bias. Targeted attention training and pre-competition psychological interventions may help improve emotion regulation and competitive performance.

## 1 Introduction

Tennis is a prototypical open-skill sport in which offense–defense transitions occur rapidly. Players must extract relevant kinematic and contextual information within a brief time window to anticipate ball trajectory and opponents’ intentions (Harris et al., 2021). During adolescence, sport-specific skills are developing rapidly, whereas self-concept and emotion regulation are still maturing. Accordingly, adolescent athletes are particularly vulnerable to pre-competition anxiety, especially in socio-evaluative environments (e.g., coaches, parents, peers, and spectators) (Beenen et al., 2025; Gwyther et al., 2024; Walton et al., 2024; Breslin et al., 2022; Cosh et al., 2024; Prior et al., 2022).

In competitive settings, anxiety is commonly distinguished into state anxiety and trait anxiety (Reardon et al., 2024). State anxiety reflects transient tension elicited by a specific match, whereas competitive trait anxiety represents a relatively stable tendency to appraise competition as threatening and to experience persistent worry and heightened vigilance over time (Domínguez-González et al., 2024; Yang et al., 2024). Sustained competitive trait anxiety has been linked to maladaptive outcomes such as burnout, reduced enjoyment, and withdrawal intentions, and it may shape how athletes allocate attentional resources when pressure is high (Tang et al., 2022; Yang et al., 2024).

Attentional Control Theory (ACT) offers a parsimonious account of how anxiety influences cognition and performance. ACT proposes that anxiety weakens top-down, goal-directed control while strengthening bottom-up, stimulus-driven processing, thereby reducing processing efficiency—particularly for inhibition and shifting functions (Eysenck et al., 2007; Eysenck et al., 2023; Sun and Zhang 2021). In sport contexts, evidence also supports ACT-consistent effects of anxiety on motor planning and control efficiency when emotional distractors are present (Coombes et al., 2009), and broader syntheses highlight the importance of sustaining goal-directed attention and inhibiting interference to perform well under pressure.

At a more fine-grained level, attentional bias to emotional information provides an observable bridge between anxiety and attentional control. Such bias can be decomposed into components, including facilitated orienting to emotional cues and difficulty disengaging from them (Folz et al., 2024). Research indicates that highly anxious individuals tend to prioritize threat-related socio-emotional signals (e.g., negative facial expressions), which can manifest as altered orienting, disengagement difficulty, or sustained allocation of attention (Blauth and Iffland, 2024; Clauss et al., 2022; Williams, 2024; Byrne et al., 2024; Lazarov et al., 2021). Because emotional facial expressions are highly salient socio-evaluative signals in real competitions, an emotional-face dot-probe task is well-suited to quantify these components in adolescent athletes; however, traditional dot-probe indices may be noisy, underscoring the value of component-based measures and careful interpretation (Shamai-Leshem et al., 2023).

Despite extensive evidence in clinical and general populations, research in competitive sport—particularly in adolescent tennis—remains limited in two respects. First, existing athlete studies have often emphasized executive-control tasks (e.g., Stroop or antisaccade) and have less frequently tested attentional bias to socio-emotional cues that athletes routinely encounter (Coombes et al., 2009; Zhang 2015). Second, many dot-probe studies rely on a single global bias score, even though component-based indices (orienting vs. disengagement) may provide more informative markers of anxiety-related processing differences and better align with contemporary attention-bias models (Folz et al., 2024; Shamai-Leshem et al., 2023). Moreover, it remains unclear which dimensions of competitive trait anxiety (e.g., social-evaluative concerns) are most strongly linked to specific attentional-bias components in adolescent tennis players, given the documented salience of socio-evaluative pressure in elite youth athletes’ mental-health risk profiles (Gwyther et al., 2024; Walton et al., 2024).

To address these gaps, the present study (a) characterized competitive trait anxiety across demographic and training variables (gender, age, training years, and sport level) in adolescent tennis players, and (b) used questionnaire screening to form high and low-anxiety groups and an emotional-face modified dot-probe task to test group differences in attentional-bias components. We computed indices of attentional orienting and difficulty disengaging from positive and negative faces and further examined correlations and regression models linking anxiety dimensions to attentional-bias indices. By focusing on component-level markers and socio-evaluative cues, the study aims to clarify the anxiety–attention pathway in adolescent tennis and to inform targeted attention training and pre-competition psychological interventions (Hang et al., 2021).

Tennis, as an open-skill sport, provides a particularly valuable context for examining the relationship between competitive trait anxiety and attentional bias. Unlike closed-skill sports, where tasks are more predictable, tennis requires athletes to make quick decisions based on dynamic and often unpredictable cues. The high visibility of performance outcomes (e.g., double faults, unforced errors) and the strong emphasis on individual achievement create a unique form of socio-evaluative pressure. Moreover, emotional facial expressions—whether from opponents, coaches, or spectators—are immediate, visible cues that carry significant socio-evaluative weight in competitive tennis. These factors make tennis an ideal context to investigate how anxiety may influence attentional processes, especially in relation to negative socio-evaluative cues.

To strengthen the theoretical rationale, we integrate ACT with a component account of attentional bias. ACT posits that competitive anxiety impairs goal-directed (top-down) attentional control and increases the influence of stimulus-driven processing, particularly for inhibition and shifting (Eysenck et al., 2007; Eysenck et al., 2023). Within contemporary attention-bias models, these control limitations should be expressed not only in overall bias scores but, more specifically, in separable components of attention allocation—facilitated orienting toward salient cues and difficulty disengaging from them (Folz et al., 2024). In socio-evaluative sport settings, emotional faces constitute highly salient signals; thus, athletes with higher competitive trait anxiety should show a stronger bias to negative faces, especially manifested as prolonged maintenance and difficulty disengaging from negative cues rather than a uniform increase in initial orienting. Accordingly, we expected that (H1) high-anxiety athletes would exhibit greater difficulty disengaging from negative faces relative to low-anxiety athletes; and (H2) the social-expectation dimension of competitive trait anxiety would be most strongly associated with, and predict, negative-cue disengagement difficulty, consistent with the centrality of perceived social evaluation in youth athletes’ anxiety profiles (Gwyther et al., 2024; Walton et al., 2024).

## 2 Experiment 1: emotional attentional bias in high *vs.* low-anxiety athletes

### 2.1 Method

#### 2.1.1 Participants

A total of 120 adolescent tennis players (aged 14–18 years) were recruited via convenience sampling from a youth tennis championship held in Hunan Province in 2020. All participants were right-handed, had normal or corrected-to-normal vision, and reported no history of neurological or psychiatric disorders. The study protocol was registered with the school ethics committee at the competition site, and written informed consent was obtained from both participants and their legal guardians.

In the main experiment, 48 adolescent tennis players from the screening sample (age range: 14–18 years; M = 16.33, SD = 1.36) were assigned to a high competitive trait anxiety group (*n* = 24) or a low competitive trait anxiety group (*n* = 24). The high-anxiety group comprised 13 males (54.2%) and 11 females (45.8%), with ages 14 years (*n* = 4), 15 years (*n* = 7), 16 years (*n* = 6), 17 years (*n* = 2), and 18 years (*n* = 5) (M = 15.88, SD = 1.39). Regarding sport level, 4 athletes (16.7%) were classified as Level II or above and 20 (83.3%) had no official sport level; training experience was 1–4 years (*n* = 6), 5–9 years (*n* = 15), and ≥9 years (*n* = 3). The low-anxiety group comprised 15 males (62.5%) and 9 females (37.5%), with ages 14 years (*n* = 1), 15 years (*n* = 3), 16 years (*n* = 4), 17 years (*n* = 8), and 18 years (*n* = 8) (M = 16.79, SD = 1.18). Sport level was Level II or above for 14 athletes (58.3%) and absent for 10 (41.7%); training experience was 1–4 years (*n* = 2), 5–9 years (*n* = 15), and ≥9 years (*n* = 7).

Competitive trait anxiety was assessed using the Pre-Competition Emotion Scale for Tennis (PES-T). Participants were rank-ordered by the PES-T total score, and those scoring in the top 20% (*n* = 24) and bottom 20% (*n* = 24) were selected to form the high-anxiety and low-anxiety groups, respectively (total *N* = 48). To minimize potential confounding effects of short-term competition schedules, the selected athletes completed the PES-T again immediately prior to the laboratory session; no significant difference emerged between the two administrations, suggesting that trait-like anxiety levels were stable across the study period. The two groups did not differ significantly in gender distribution, age, training years, or athletic level (ps > 0.05), indicating adequate group matching on key background variables.

An independent sample of 20 volunteer raters (university students and/or tennis-specialized students; approximately balanced by sex) participated in the stimulus pre-rating procedure. They received course credit or modest compensation.

#### 2.1.2 Materials

##### 2.1.2.1 Competitive trait anxiety (PES-T)

Competitive trait anxiety was measured using the Chinese version of the Pre-Competition Emotion Scale for Tennis (PES-T). The scale comprises four subscales: personal failure anxiety, social expectation anxiety, somatic anxiety, and self-confidence (reverse-scored). Items are rated on a 5-point Likert scale, with higher total scores indicating higher competitive trait anxiety. In the present sample, Cronbach’s *α* values for the four subscales were all above 0.85, and the total-scale α was approximately 0.90, indicating good internal consistency.

##### 2.1.2.2 Emotional face stimuli

**Figure 1:**
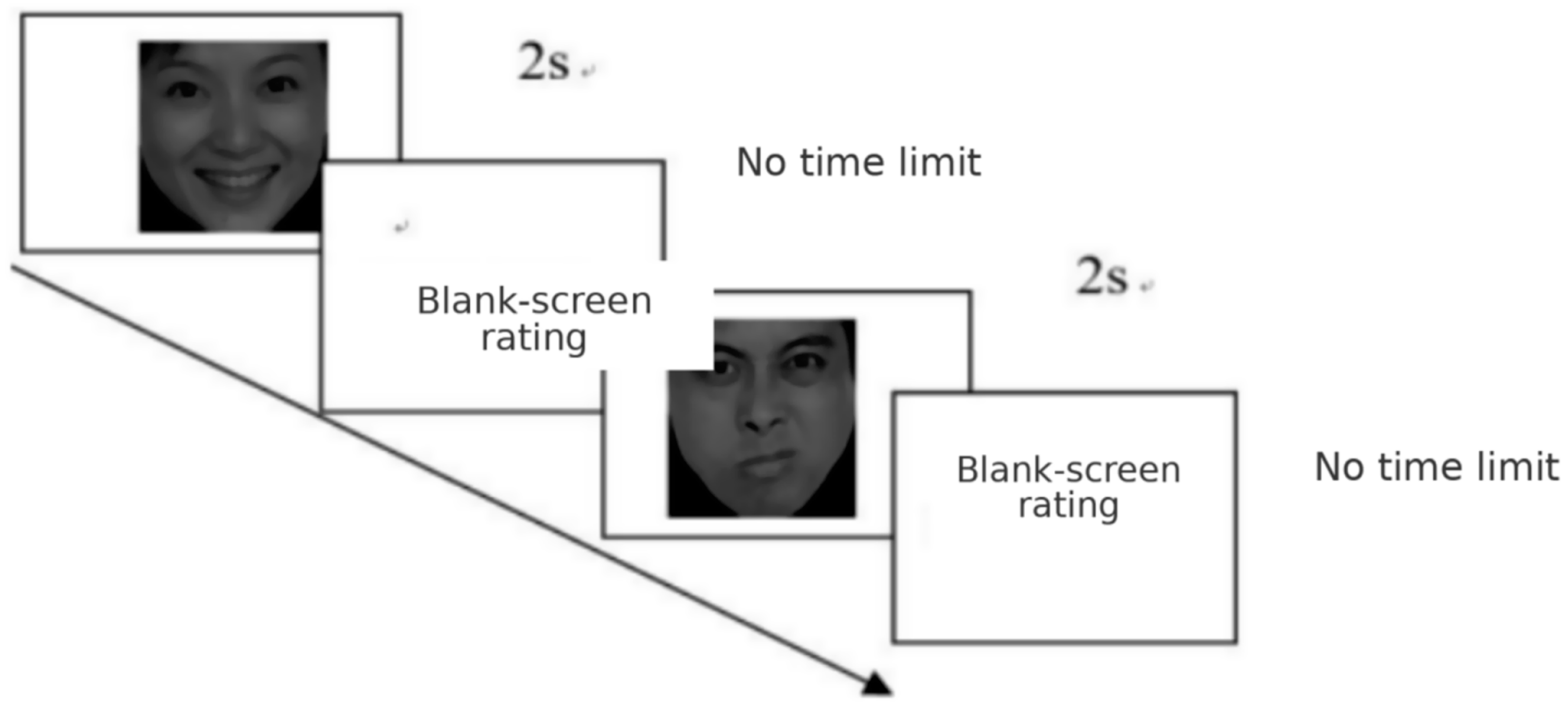
Trial flowchart for the stimulus (image) evaluation task (images reproduced with permission from ‘The Chinese Facial Affective Picture System [CFAPS]’, Shenzhen Naozhi Development Technology Co., Ltd., Gong et al., 2011).

Face stimuli were drawn from the Chinese Facial Affective Picture System. An initial pool included approximately 80 negative faces (e.g., anger, sadness, disgust), 80 positive faces (e.g., happiness), and 90 neutral faces. All images were front-facing, resized to a uniform format, and edited to remove background and non-facial information (Figure 1).

During the pre-rating phase, 20 raters evaluated each face on 9-point scales for valence (1 = very unpleasant, 9 = very pleasant) and arousal (1 = very calm, 9 = very arousing). Based on mean ratings, 40 faces per category were selected: negative faces with low valence (≈1–3) and high arousal (≈7–9), positive faces with high valence (≈7–9) and high arousal (≈7–9), and neutral faces with mid-range valence/arousal (≈4–6). Each category contained 20 male and 20 female faces. One-way ANOVAs confirmed strong category differences. Valence differed significantly across negative (*M* = 2.18, *SD* = 0.67), positive (*M* = 8.10, *SD* = 0.67), and neutral faces (*M* = 4.39, *SD* = 0.33), *F*(2, 137) = 1190.52, *p* < 0.001, *ηp^2^* = 0.946. Arousal also differed across categories (negative: *M* = 8.21, *SD* = 0.51; positive: *M* = 8.19, *SD* = 0.63; neutral: *M* = 4.39, *SD* = 0.34), *F*(2, 137) = 1065.44, *p* < 0.001, *ηp^2^* = 0.940. Bonferroni-corrected pairwise tests indicated that positive > neutral > negative for valence (all *ps* < 0.001); for arousal, negative and positive faces were rated as more arousing than neutral faces (*ps* < 0.001), whereas negative and positive faces did not differ (*p* = 0.845).

#### 2.1.3 Apparatus

The experiment was conducted in a quiet laboratory with appropriate lighting. Stimuli were presented on a 15.6-inch laptop computer at a viewing distance of approximately 60 cm. The task was programmed in E-Prime 2.0 to control stimulus timing and to record response accuracy and reaction time (RT).

#### 2.1.4 Procedure

The study used a two-stage screening plus one-stage laboratory assessment design: (a) questionnaire screening in the natural pre-competition context to identify high and low-anxiety athletes, followed by (b) a laboratory dot-probe task to assess emotional attentional bias. Stimulus pre-rating was completed prior to the main experiment and served only for stimulus selection.

##### 2.1.4.1 Questionnaire administration and group selection

Two to three days before the championship began, the PES-T was administered in a classroom setting organized by the training units. Standardized instructions emphasized that there were no “right” or “wrong” answers and that responses should reflect genuine feelings. The survey required approximately 20–30 min to complete and was checked for completeness on-site. Participants were then rank-ordered by PES-T total score to select the high and low-anxiety groups for the laboratory session.

##### 2.1.4.2 Dot-probe task (attentional bias assessment)

**Figure 2:**
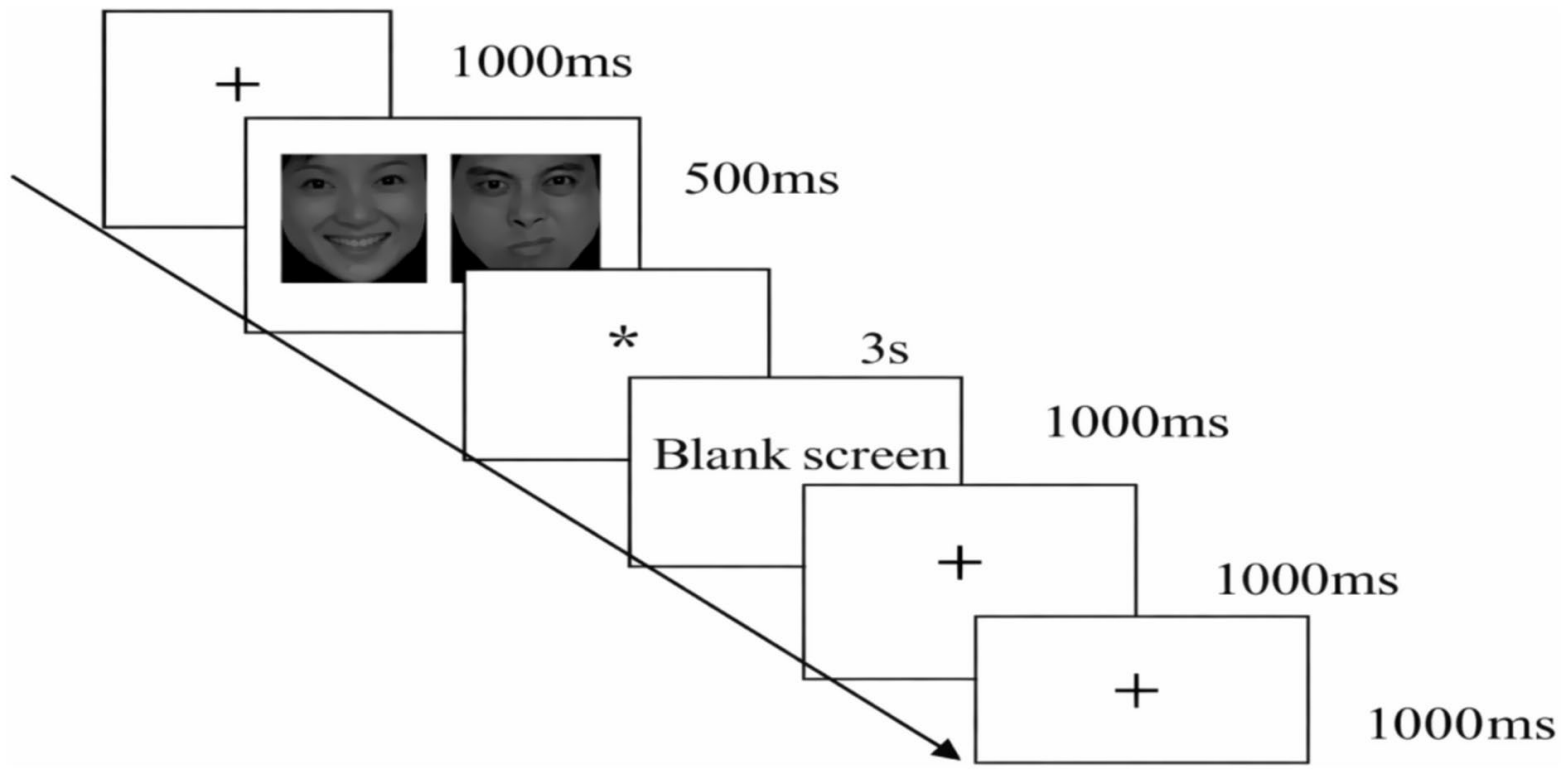
Trial flowchart for a single attentional-bias task trial (images reproduced with permission from ‘The Chinese Facial Affective Picture System [CFAPS]’, Shenzhen Naozhi Development Technology Co., Ltd., Gong et al., 2011).

Participants were tested individually. Each trial began with a central fixation cross (“+”) presented for 1,000 ms. Next, a pair of faces appeared simultaneously to the left and right of fixation for 500 ms. Face-pair types included negative–neutral, positive–neutral, and neutral–neutral. Immediately after face offset, a probe (“*”) appeared in the location of either the left or right face. Participants were instructed to respond as quickly and accurately as possible by pressing “S” if the probe appeared on the left and “K” if it appeared on the right. The probe remained on screen until a response was made or a maximum duration of 3,000 ms elapsed. An inter-trial blank screen was then presented for 1,000 ms. Participants completed ~10 practice trials (with additional practice provided if needed) before the 120-trial formal task (Figure 2).

In the emotion–neutral pair conditions, the emotional face (negative or positive) appeared equally often on the left and right. Probe location was randomized and counterbalanced such that, within each emotion–neutral condition, probes appeared equally often on the same side as the emotional face (congruent trials) and on the same side as the neutral face (incongruent trials). In the neutral–neutral condition, probe location was randomized across left/right. Trial order was randomized for each participant. Participants were instructed to maintain central fixation, keep their head stable, and minimize blinking and body movement during the task to reduce noise.

#### 2.1.5 Data reduction and statistical analysis

##### 2.1.5.1 Preprocessing

Trials with incorrect responses were excluded. For correct trials, responses with RTs < 200 ms or > 1,000 ms were removed as invalid. Mean RTs were then computed for each participant in each condition (Emotion Type × Congruency). Across participants, approximately 3% of trials were excluded in total (incorrect responses: ~2%; RTs outside 200–1,000 ms: ~1%), indicating overall good data quality.

##### 2.1.5.2 Attentional bias indices

The calculation of attentional bias indices in this study follows the component-based approach, which decomposes attentional bias into distinct phases, such as initial orienting and difficulty disengaging from emotional cues. This approach is grounded in Attentional Control Theory (ACT), which posits that anxiety impairs top-down control over attention, particularly in response to emotional stimuli (Eysenck et al., 2007; Eysenck et al., 2023). The specific calculation of attentional bias indices involves measuring response times (RT) in the dot-probe task and computing bias scores based on the differential RTs when emotional faces (positive, negative, neutral) are presented. This method has been widely used and validated in both clinical and sport psychology research (Clauss et al., 2022; Shamai-Leshem et al., 2023).

Following prior work, attentional bias was decomposed into overall bias, orienting, and disengagement components. Using condition means from emotion–neutral and neutral–neutral trials, the following indices were computed for each emotion type:

###### 2.1.5.2.1 Bias score (overall attentional bias)

Bias = RT_incongruent − RT_congruent, where RT_congruent is the mean RT when the probe replaces the emotional face and RT_incongruent is the mean RT when the probe replaces the neutral face. Bias > 0 indicates faster responding to probes at the emotional-face location (i.e., attentional bias toward that emotion).

###### 2.1.5.2.2 Orienting (facilitated initial orienting)

Orienting = RT_neutral–neutral − RT_emotion–neutral, where RT_emotion–neutral is the mean RT collapsed across probe positions in emotion–neutral trials. Orienting > 0 indicates faster responses in emotion–neutral versus neutral–neutral trials.

###### 2.1.5.2.3 Disengagement (difficulty disengaging from emotional stimuli)

Disengagement = RT_neutral–emotion − RT_neutral–neutral, where RT_neutral–emotion is the mean RT when the probe appears at the neutral-face location while the other side contains an emotional face. Disengagement > 0 indicates slower responding relative to neutral–neutral trials, consistent with difficulty disengaging attention from emotional cues.

### 2.2 Results

#### 2.2.1 Competitive trait anxiety in adolescent tennis players

##### 2.2.1.1 Gender differences

An independent-samples *t* test indicated that female athletes (M = 38.39, SD = 10.44) reported higher competitive trait anxiety than male athletes (M = 33.66, SD = 12.20), *t*(118) = −2.22, *p* = 0.028, *r* = −0.20 (*η*^2^ = 0.04) (see Table 1).

**Table 1:** Competitive trait anxiety levels in adolescent tennis players by gender (*N* = 120).

Gender	Competitive trait anxiety (M ± SD)	*t*	*p*
Male	33.66 ± 12.20		
Female	38.39 ± 10.44	−2.22	0.028

##### 2.2.1.2 Age differences

A one-way ANOVA revealed a significant effect of age on competitive trait anxiety (see Table 2), *F*(4, 115) = 5.40, *p* = 0.001, *ηp*^2^ = 0.158. Descriptively, anxiety scores decreased with increasing age (14 years: M = 40.80, SD = 11.58; 15 years: M = 40.29, SD = 9.34; 16 years: M = 37.23, SD = 10.85; 17 years: M = 30.77, SD = 10.71; 18 years: M = 29.35, SD = 12.44). Post-hoc pairwise comparisons indicated that the 17–18-year-old group scored significantly lower than several younger age groups (see Table 3).

**Table 2:** Competitive trait anxiety levels in adolescent tennis players across age groups (M ± SD).

Age (years)	Competitive trait anxiety (M ± SD)	*F*	*p*
14	40.80 ± 11.58		
15	40.29 ± 9.34		
16	37.23 ± 10.85		
17	30.77 ± 10.71		
18	29.35 ± 12.44	5.40	0.001

**Table 3:** *Post hoc* pairwise comparisons of competitive trait anxiety among age groups.

Comparison (I–J)	Test statistic	Standard error	*p*
Age18-Age15	34.26	10.34	0.001
Age17-Age15	31.18	9.55	0.001
Age16-Age15	11.22	9.31	0.220
Age14-Age15	−3.02	10.84	0.780
Age18-Age14	31.24	11.46	0.006
Age17-Age14	28.16	10.76	0.009
Age16-Age14	8.20	10.35	0.430
Age18-Age16	23.03	10.03	0.020
Age17-Age16	19.95	9.22	0.030

##### 2.2.1.3 Training-years differences

Competitive trait anxiety differed significantly across training-years groups (see Table 4), *F*(2, 117) = 11.50, *p* < 0.001, *ηp*^2^ = 0.164. Athletes with longer training histories reported lower anxiety (1–4 years: M = 43.41, SD = 12.20; 5–9 years: M = 35.26, SD = 10.04; ≥9 years: M = 27.80, SD = 11.95). Post-hoc tests showed significant differences between all pairs of training-years groups (see Table 5).

**Table 4:** Competitive trait anxiety levels in adolescent tennis players across training-years groups (M ± SD).

Training years	Competitive trait anxiety (M ± SD)	*F*	*p*
1–4 Years	43.41 ± 12.20		
5–9 Years	35.26 ± 10.04		
≥9 Years	27.80 ± 11.95	11.50	< 0.001

**Table 5:** Post hoc pairwise comparisons of competitive trait anxiety among training-years groups.

Comparison (I–J)	Test statistic	Standard error	*p*
≥ 9 years – 5–9 years	21.16	8.73	0.015
≥ 9 years – 1–4 years	47.48	10.52	< 0.001
5–9 years – 1–4 years	26.32	8.14	0.001

##### 2.2.1.4 Sport-level differences

Athletes with National Level 2 or above (M = 25.55, SD = 11.61) reported significantly lower competitive trait anxiety than athletes without an official level (M = 37.93, SD = 10.49), *t*(118) = −5.14, *p* < 0.001, *r* = −0.43 (*η*^2^ = 0.18)(see Table 6).

**Table 6:** Competitive trait anxiety levels in adolescent tennis players by sport level (M ± SD).

Sport level	Competitive trait anxiety (M ± SD)	*t*	*p*
National level 2 or above	25.55 ± 11.61		
No official level	37.93 ± 10.49	−5.14	< 0.001

#### 2.2.2 Attentional bias in high *vs.* low-anxiety groups

##### 2.2.2.1 Reaction time (RT) analysis

RTs were submitted to a 2 (Group: high vs. low competitive trait anxiety) × 2 (Valence: negative vs. positive) × 2 (Probe location: congruent vs. incongruent) mixed ANOVA (see Table 7, Table 8). The main effect of Group was significant, *F*(1, 45) = 13.879, *p* = 0.009, *ηp*^2^ = 0.236, as was the main effect of Probe location, *F*(1, 45) = 4.983, *p* = 0.026, *ηp*^2^ = 0.100. Critically, the Valence × Group interaction was significant, *F*(1, 45) = 12.326, *p* = 0.012, *ηp*^2^ = 0.215; other effects were not significant (ps > 0.05).

**Table 7:** Reaction times (ms) for the high and low-competitive trait anxiety groups by emotion condition and probe location (M ± SD).

Probe location	Image type	High competitive trait anxiety (*n* = 24)	Low competitive trait anxiety (*n* = 24)
Congruent	Negative–Neutral	399.11 ± 89.07	416.64 ± 91.33
Congruent	Positive–Neutral	400.33 ± 68.68	393.63 ± 81.90
Incongruent	Negative–Neutral	414.52 ± 98.80	415.49 ± 89.28
Incongruent	Positive–Neutral	397.79 ± 88.08	415.23 ± 97.54
	Neutral–Neutral	393.87 ± 81.33	409.59 ± 83.61

**Table 8:** Results of the mixed-design ANOVA for reaction times.

Source of variation	df1	df2	*F*	*p*
Image type	1	45	1.387	0.239
Group	1	45	13.879	0.009
Probe location	1	45	4.983	0.026
Image type × group	1	45	12.326	0.012
Image type × probe Location	1	45	0.024	0.878
Group × probe location	1	45	0.688	0.407
Image type × group × probe location	1	45	3.250	0.072

Simple-effects tests indicated that, in the high-anxiety group, RTs were faster for congruent than incongruent probes in the negative–neutral condition (*F*(1, 22) = 13.61, *p* < 0.05, dz. = −0.77). In the low-anxiety group, RTs were faster for congruent than incongruent probes in the positive–neutral condition (*F*(1, 21) = 3.80, *p* < 0.05, dz. = −0.42). Descriptively, the high-anxiety group showed a location advantage for negative cues (negative–neutral: congruent M = 399.11 ms, SD = 89.07; incongruent M = 414.52 ms, SD = 98.80), whereas the low-anxiety group showed a location advantage for positive cues (positive–neutral: congruent M = 393.63 ms, SD = 81.90; incongruent M = 415.23 ms, SD = 97.54).

##### 2.2.2.2 Component indices (bias, orienting, disengagement)

Bias, orienting, and disengagement indices were computed and tested against zero using one-sample t tests (see Tables 9–11). In the high-anxiety group, the bias score for negative faces was significantly greater than zero (M = 14.41, SD = 9.73), *t*(22) = 1.984, *p* = 0.049, dz. = 0.41. The disengagement score for negative faces was also significantly greater than zero (M = 20.64, SD = 17.47), *t*(22) = 2.555, *p* = 0.011, dz. = 0.53; other indices were not significant. In the low-anxiety group, the bias score for positive faces was significantly greater than zero (M = 21.60, SD = 14.24), *t*(21) = 2.244, *p* = 0.026, dz. = 0.48; all remaining tests were nonsignificant.

**Table 9:** Component indices of attentional bias by emotion condition for the high and low-competitive trait anxiety groups (M ± SD).

Attentional index	Image type	High competitive trait anxiety (*n* = 24)	Low competitive trait anxiety (*n* = 24)
Bias score	Negative	14.41 ± 9.73	−1.15 ± 9.63
Bias score	Positive	−2.53 ± 11.23	21.60 ± 14.24
Facilitated orienting	Negative	−5.32 ± 7.74	−8.92 ± 1.16
Facilitated orienting	Positive	−6.46 ± 12.22	14.09 ± 13.93
Difficulty disengaging	Negative	20.64 ± 17.47	7.77 ± 25.12
Difficulty disengaging	Positive	3.92 ± 11.11	7.77 ± 11.42

**Table 10:** One-sample *t* tests of attentional indices in the high-competitive trait anxiety group (*n* = 24; test value = 0).

Attentional index	Image type	*t*	*p*
Bias score	Negative	1.984	0.049
Bias score	Positive	−0.333	0.740
Facilitated orienting	Negative	−0.676	0.500
Facilitated orienting	Positive	−0.935	0.351
Difficulty disengaging	Negative	2.555	0.011
Difficulty disengaging	Positive	0.522	0.602

**Table 11:** One-sample t tests of attentional indices in the low-competitive trait anxiety group (*n* = 24; test value = 0).

Attentional Index	Image type	*t*	*p*
Bias score	Negative	−0.177	0.860
Bias score	Positive	2.244	0.026
Facilitated orienting	Negative	−1.134	0.258
Facilitated orienting	Positive	1.479	0.140
Difficulty disengaging	Negative	1.006	0.315
Difficulty disengaging	Positive	0.934	0.351

#### 2.2.3 Associations between anxiety dimensions and attentional bias

##### 2.2.3.1 Correlation

Within the high-anxiety group, Pearson correlations showed that social-expectation anxiety was positively associated with disengagement difficulty (*r* = 0.658, *p* = 0.010; *r*^2^ = 0.433). Correlations between disengagement difficulty and both personal-failure anxiety (*r* = −0.210, *p* = 0.335) and somatic anxiety (*r* = 0.029, *p* = 0.897) were not significant (see Tables 12, 13).

**Table 12:** Scores for each dimension of competitive trait anxiety in the high-competitive trait anxiety group (*n* = 24; M ± SD).

Dimension	M ± SD
Self-confidence	14.50 ± 2.99
Somatic anxiety	19.83 ± 2.66
Social expectation anxiety	25.96 ± 2.89
Personal failure anxiety	20.58 ± 1.79

**Table 13:** Pearson correlations between anxiety dimensions and difficulty disengaging scores in the high-competitive trait anxiety group (*n* = 24).

Variables	*r*	*p*
Personal failure anxiety *vs.* difficulty disengaging	−0.210	0.335
Social expectation anxiety vs. difficulty disengaging	0.658	0.010
Somatic anxiety vs. difficulty disengaging	0.029	0.897

##### 2.2.3.2 Regression

A linear regression predicting disengagement difficulty from social-expectation anxiety was significant (see Table 14), *F* = 16.042, adjusted *R*^2^ = 0.433. Social-expectation anxiety positively predicted disengagement difficulty (*B* = 17.674, *β* = 0.658, *t* = 4.005, *p* = 0.001), with an effect size of *f*^2^ = 0.76. The fitted equation was: disengagement difficulty = −658.443 + 17.674 × (social-expectation anxiety).

**Table 14:** Linear regression predicting difficulty disengaging from social expectation anxiety in the high-competitive trait anxiety group.

Predictor	Outcome	B	*β*	*t*	*p*	Adjusted *R*^2^	*F*
Constant	Difficulty disengaging	−658.443		−3.667	0.001		
Social expectation anxiety	Difficulty disengaging	17.674	0.658	4.005	0.001	0.433	16.042

## 3 Discussion

### 3.1 Summary of key findings

In line with the study aims, the present findings converge on an anxiety–attention pathway in adolescent tennis, characterized primarily by difficulty disengaging from negative socio-evaluative cues rather than generalized vigilance across all emotional stimuli.

#### 3.1.1 Group characteristics of competitive trait anxiety

Female athletes reported higher competitive trait anxiety than male athletes. Rather than reflecting a purely sport-specific phenomenon, this pattern is consistent with broader evidence that adolescent and young female athletes may experience greater socio-evaluative concerns and perceived pressure in competition (Kew et al., 2024; Correia and Rosado, 2019). In tennis, performance is highly visible and error consequences are salient (e.g., double faults, unforced errors), which can amplify self-presentational demands. From a developmental perspective, ongoing maturation of self-concept and emotion regulation during adolescence may further increase sensitivity to evaluation, making competitive contexts particularly anxiety-eliciting for some athletes (Beenen et al., 2025). The effect size was small, suggesting substantial overlap between genders; nonetheless, it highlights the importance of considering gender as a potential moderator when designing screening and prevention efforts.

Competitive trait anxiety tended to be lower among older, more experienced, and higher-level athletes. This trend can be interpreted as a protective effect of accumulated mastery experiences, improved coping repertoires, and better expectancy calibration, which jointly reduce uncertainty and perceived threat before competition. It also aligns with accounts indicating that competitive demands interact with skill level to shape psychological load and attention-control requirements in sport (Rahimi et al., 2022). An alternative (non-exclusive) explanation is selection: athletes with persistently high anxiety may be more likely to disengage from intensive training or drop out over time, resulting in a more resilient group at higher levels. Longitudinal tracking would help disentangle developmental change from selection effects.

### 3.2 Theoretical implications

These results extend Attentional Control Theory (ACT) to an ecologically relevant sport setting by specifying which component of attentional bias is most sensitive to competitive trait anxiety in adolescents (i.e., disengagement rather than initial orienting), consistent with the notion that anxiety impairs top-down control over attention under socio-evaluative threat.

### 3.3 Attentional-bias patterns associated with anxiety level

The central finding of the attentional-bias task was that anxiety level was associated with qualitatively different processing of socio-emotional cues. Athletes in the high-anxiety group showed faster responding when probes replaced negative faces and, more importantly, a reliable difficulty disengaging from negative cues. This pattern maps closely onto Attentional Control Theory (ACT), which proposes that anxiety weakens goal-directed control and increases the influence of stimulus-driven processing, particularly for inhibitory and shifting functions (Eysenck et al., 2007; Eysenck et al., 2023). Conceptually, disengagement difficulty reflects impaired attentional shifting away from threat, which is precisely the type of control limitation ACT predicts under anxiety.

Our component-based results also converge with evidence from clinical and general populations showing that anxiety-related attentional bias is often expressed as sustained attention to threat and impaired disengagement (Clauss et al., 2022; Folz et al., 2024). In contrast, the low-anxiety group showed a bias toward positive faces, which may reflect a more adaptive, approach-oriented attentional set in evaluative contexts. Such a tendency could serve as a protective mechanism by prioritizing affiliative or encouraging signals and reducing the cognitive costs of threat monitoring, potentially supporting more efficient performance-related processing.

Notably, dot-probe RT difference scores have been criticized for limited reliability, and global bias indices can mask distinct orienting versus disengagement mechanisms (Shamai-Leshem et al., 2023). By decomposing bias into components, the present findings help clarify that the anxiety-linked signature in adolescent tennis was primarily a disengagement difficulty for negative faces rather than a generalized bias across all components. This component-level account provides a more specific target for intervention and for future measurement refinement (Xu et al., 2024).

### 3.4 Practical implications

From an applied perspective, the component-level pattern suggests that interventions may be more effective if they prioritize training attentional disengagement from negative socio-evaluative cues (e.g., brief attention-bias modification or attentional control exercises) (Rooney et al., 2024) and combine this with pre-competition routines that reduce social-evaluative worry.

Group differences were particularly clear in the present pre-competition assessment, which likely increased the salience of socio-evaluative threat cues. Emotional faces are direct social signals that mirror the real competitive environment (coaches’ reactions, opponents’ expressions, and spectators’ feedback), thereby enhancing ecological validity relative to word stimuli. This is consistent with the view that performance anxiety in young athletes is shaped by multi-layered socio-evaluative contexts (Beenen et al., 2025).

In addition, testing shortly before competition and near the competition setting may have increased the accessibility of threat appraisals and vigilance, making trait-like tendencies more observable. ACT further predicts that anxiety-related control costs become more evident as task demands and perceived stakes increase (Eysenck et al., 2007; Eysenck et al., 2023). Thus, the timing and context of assessment may partly explain why the interaction between image type and group was robust in this sample.

Finally, tennis as an open-skill sport requires continuous monitoring of dynamic cues and rapid decision-making, which can magnify the opportunity costs of sustained attention to threat. When attention is “held” by negative social cues, athletes may have fewer resources available for anticipatory processing and tactical adjustments, potentially creating a self-reinforcing anxiety–performance cycle under pressure (Rahimi et al., 2022).

### 3.5 Limitations and future directions

Because competitive context can modulate attentional and executive-control demands, future studies should more directly examine context activation (e.g., pre-competition vs. training settings) and social-evaluative pressure as moderators of attentional-bias components, and evaluate whether the same disengagement-focused signature emerges across athlete populations and performance settings (Rahimi et al., 2022).

Within the high-anxiety group, social-expectation anxiety showed the strongest association with disengagement difficulty and significantly predicted this component. This finding suggests that the anxiety–attention pathway in adolescent tennis may be especially driven by perceived social evaluation (e.g., concern about coaches’ or parents’ judgments) rather than somatic arousal or personal-failure concerns. Mechanistically, social-evaluative threat is well-positioned to increase the salience of negative facial expressions, thereby prolonging attention allocation and impairing disengagement. The specificity of this link also helps explain why other anxiety dimensions showed weaker or non-significant relations with attentional indices in the present data.

Within the high competitive trait anxiety group, social-expectation anxiety was strongly and positively correlated with negative-cue disengagement difficulty (*r* = 0.658; ~43% explained variance) and significantly predicted disengagement scores. This suggests that when athletes worry more about failing to meet others’ expectations, they may find it harder to disengage attention from potentially negative social signals, thereby maintaining a negative bias and sustaining pre-competition anxiety. Building on early evidence that attention-bias modification can benefit athletes (Zhao et al., 2025), future interventions could integrate attention training under social-evaluative contexts with cognitive reappraisal and self-compassion strategies to more directly target the “social expectation → threat appraisal → disengagement difficulty” maintenance loop (Li et al., 2025; Wang et al., 2025).

Future work should test whether the present component-level pattern generalizes across different sport types and levels of socio-evaluative threat by manipulating competitive context (e.g., audience/coach presence, ranking stakes) and by sampling both open and closed-skill sports.

At the attentional level, training protocols that explicitly practice shifting attention away from negative faces and toward task-relevant cues may be promising. Attention-bias modification or gaze-control training can be integrated into per iodized preparation, with progress monitored using component-based indices rather than a single global score (Shamai-Leshem et al., 2023; Xu et al., 2024).

Several limitations should be acknowledged. First, the design was cross-sectional, limiting causal inference; future work should test whether reducing social-expectation anxiety or improving disengagement leads to downstream performance benefits. Second, reliance on RT measures alone may underestimate attentional dynamics; combining eye tracking, EEG/ERP, or neuroimaging with behavioral indices would improve construct validity and help resolve measurement noise concerns (Clauss et al., 2022; Shamai-Leshem et al., 2023; Lazarov et al., 2021). Third, the sample was drawn from a specific training context; replication across competitive levels and regions would strengthen generalizability.

Given concerns about dot-probe reliability (Xu et al., 2024), future studies should combine multiple indicators (e.g., eye-tracking indices such as dwell time/first fixation, or computational modeling) to better differentiate orienting versus maintenance/disengagement components. In addition, larger samples and more refined situational manipulations (e.g., explicit social-evaluative threat, different pre-competition time points) would help test how “context activation” moderates bias expression and whether patterns differ between open and closed-skill sports.

## 4 Conclusion

Overall, the present study provides evidence that competitive trait anxiety in adolescent tennis players is most strongly reflected in difficulty disengaging from negative facial cues, and that social-expectation anxiety is a key correlate of this processing pattern. This component-level account helps clarify mechanisms and informs targeted psychological preparation for youth athletes.

## Data Availability

The raw data supporting the conclusions of this article will be made available by the authors, without undue reservation.
